# Evaluation of the Potential Use of a Collagen-Based Protein Hydrolysate as a Plant Multi-Stress Protectant

**DOI:** 10.3389/fpls.2021.600623

**Published:** 2021-02-09

**Authors:** Stefano Ambrosini, Davide Sega, Chiara Santi, Anita Zamboni, Zeno Varanini, Tiziana Pandolfini

**Affiliations:** Dipartimento di Biotecnologie, Università degli Studi di Verona, Verona, Italy

**Keywords:** protein hydrolysates, biostimulants, collagen, crop performance, Fe deprivation, abiotic stress

## Abstract

Protein hydrolysates (PHs) are a class of plant biostimulants used in the agricultural practice to improve crop performance. In this study, we have assessed the capacity of a commercial PH derived from bovine collagen to mitigate drought, hypoxic, and Fe deficiency stress in *Zea mays*. As for the drought and hypoxic stresses, hydroponically grown plants treated with the PH exhibited an increased growth and absorption area of the roots compared with those treated with inorganic nitrogen. In the case of Fe deficiency, plants supplied with the PH mixed with FeCl_3_ showed a faster recovery from deficiency compared to plants supplied with FeCl_3_ alone or with FeEDTA, resulting in higher SPAD values, a greater concentration of Fe in the leaves and modulation in the expression of genes related to Fe. Moreover, through the analysis of circular dichroism spectra, we assessed that the PH interacts with Fe in a dose-dependent manner. Various hypothesis about the mechanisms of action of the collagen-based PH as stress protectant particularly in Fe-deficiency, are discussed.

## Introduction

Ever since their appearance on land, plants had to deal with numerous abiotic stresses, including low or high temperature, deficient or excessive water, high light intensity and ultraviolet (UV) radiation, nutrient shortages or imbalances. These stresses are accountable for great crop yield loss (Wang et al., 2004; Wani et al., 2016) and are exacerbated by recent climate changes (Boyer, 1982; Rahmstorf and Coumou, 2011). The negative impact on plant growth is worsened when these stresses are combined, for instance, drought and heat stress were responsible for as much as the 40% of reduction in crops yield for maize (Daryanto et al., 2016), and up to 68% for cowpea (Farooq et al., 2017). Moreover, water shortage and soil salinity trigger oxidative, osmotic and temperature stress, representing another major challenge for productivity (Landi et al., 2017). In the future, the dramatic increase in land degradation rate is expected to result in a drastic loss of planet arable lands (Scherr, 1999). According to the data published by the European Union in the World Atlas of Desertification, the combined effects of soil degradation and climate changes will result in 10% reduction of global crop yield by 2050, with severe economic losses (Cherlet et al., 2018). The future scenarios urge scientists to search for new technologies to improve crop productivity, focusing on organic substances, which not only could replace chemical fertilizers, often responsible for environmental pollution, but could also be exploited to contrast abiotic stresses (Suhag, 2016). Nowadays, an increasingly employed resource in agriculture are plant biostimulants. This term refers to a very broad class of substances and microorganisms that includes – as defined by the European Union – “fertilizing products the function of which is to stimulate plant nutrition processes independently of the products’ nutrient content with the sole aim of improving one or more of the following characteristics of the plant or the plant rhizosphere: (i) nutrient use efficiency, (ii) tolerance to abiotic stress, (iii) quality traits, or (iv) availability of confined nutrients in the soil or rhizosphere” (European Union, 2019). Many diverse bioactive substances are catalogued as plant biostimulants based on their agricultural function claims, including: (i) humic and fulvic acids, (ii) animal and vegetal protein hydrolysates (PHs), (iii) macroalgae seaweeds extracts, and (iv) silicon, and also some beneficial microorganisms such as (i) arbuscular mycorrhizal fungi (AMF) and (ii) nitrogen-fixing bacteria of strains belonging to the genera *Rhizobium*, *Azotobacter*, and *Azospirillum*. Among the listed categories, protein hydrolysates are particularly interesting, not only because they are effective biostimulants, but especially because they are mainly produced from organic waste, well fitting into the eco-friendly scheme of circular economy (Xu and Geelen, 2018). PHs are by-products from several industrial activities and can either have animal- or plant- origins. Some of the animal matrix from which PHs can be obtained are epithelial or connective tissues, hen feathers, and bone meal (Drobek et al., 2019), while plant-derived PHs can come from carob germ proteins, alfalfa residue, wheat-condensed distiller solubles, *Nicotiana* cell wall glycoproteins and algal proteins (Calvo et al., 2014).

Most of the studies regarding PHs highlight their capacity to improve plant growth and nutrient use efficiency, proposing some mechanisms of action which might include the hormone-like activities of bioactive peptides (Santi et al., 2017; Ertani et al., 2019), the stimulation of carbon and nitrogen metabolism, as well as the regulation of nutrient uptake (Ertani et al., 2009; Colla et al., 2014). For instance, Santi et al. proved that the treatment with a bovine collagen-derived PH remarkably enhanced maize root increasing the root accumulation of K, Zn, Cu, and Mn when compared with inorganic N and amino acids treatments (Santi et al., 2017). Moreover, in the same work, the microarray analysis suggested that the treatment with the bovine collagen-derived PH to the nutrient solution caused major changes in the transcriptional profile of the roots, altering the expression of genes involved in the cell wall organization, the transport processes, the stress responses and the hormone metabolism. Recently, Paul et al. (2019b) tested eight different plant-derived protein hydrolysates on tomato plants, reporting, for some of the treatments, an increase in relative growth rate and growth performance. Moreover, metabolomic profiles of the plants treated with PHs showed that ethylene precursor 1-aminocyclopropane-1-carboxylate (ACC) and polyamines were positively modulated, and the ROS-mediated signaling pathways were affected as well (Paul et al., 2019b).

While there is a vast literature on PHs efficacy as plant growth and nutrient use efficiency enhancer, relatively few works have tried to demonstrate that these compounds are also capable to ameliorate plant performances under abiotic stress conditions. In an interesting paper, Ertani et al. proved that a plant-derived PH increased plant biomass in salt-stressed maize plants (Ertani et al., 2012), ascribing the beneficial effects to the triacontanol, a fatty acid known to act as plant growth regulator (Chen et al., 2002) and salt-stress mitigator (Çavuşoğlu et al., 2008), which is present in the PH in a noticeable amount. Another study from Lucini et al. (2015) demonstrated the beneficial effects of a plant-derived PH treatment on lettuce plants grown in pots under saline stress. The PH treatment helped the plants to better respond to saline environment compared to those treated with water only, showing an increased shoot fresh yield. In this case the authors suggested that the PH mitigated the stress by attenuating the oxidative stress, improving osmotic adjustment, and rewiring the hormone, sterol, terpene and glucosinolate profiles (Lucini et al., 2015). More recently, the work of Trevisan et al. (2019) investigated the activity of a collagen-derived PH as stress-mitigator, in maize seedlings subjected to hypoxic, saline and nutritional stress. The plants treated with this biostimulant produced longer roots in all the three stress conditions than those which did not receive the treatment. The transcription level of the superoxide dismutase *ZmSOD1a* gene was induced upon PH treatment in hypoxia, nutritional deficiency, and when saline and nutritional stress were combined. The authors therefore suggested that the PH might mitigate the effects of different stresses by regulating of the expression of genes involved in ROS scavenging (Trevisan et al., 2019). Another recent work, from Paul et al. (2019a), positively assessed the beneficial effect of a plant-derived PH as stress-mitigator on tomato plants grown under limited water availability. They suggested that PH-treated plants were able to better cope with oxidative stress thanks to major changes in phytohormones and lipid profiles (Paul et al., 2019a). Lastly, Casadesús et al. (2019, 2020) assessed the beneficial effects of a product derived from enzymatic hydrolysis of animal proteins in tomato plants grown under water, temperature or nutrient stress suggesting that the positive effects on root growth are due to the increased biosynthesis of specific phytohormones (Casadesús et al., 2019, 2020).

The aim of this work is to investigate the capacity of a collagen-based PH to act as multi-stress protectant when supplied to plant species of agricultural interest by setting up fast and reliable experimental laboratory tests. We carried out the experiments on hydroponically grown maize plants treated with a commercial PH (LMW10 – produced by SICIT group) derived from acid hydrolysis of bovine collagen. We demonstrated that the PH was able to protect the root apparatus from drought and hypoxic stresses. We also proved that the PH quickened the recovery of plants after iron (Fe) deficiency. Moreover, we provide evidence of the chemical interaction between the PH and Fe ions as a possible mechanism that could favor the micronutrient availability for root uptake in the nutrient solution.

## Materials and Methods

### Plant Materials, Growth Conditions and Phenotypic Analyses

Maize seeds (P0943 Hybrid, Pioneer Italia S.p.A.) were soaked in water for 24 h and germinated in the dark on wet filter paper for 72 h. Seedlings with similar size were then transferred in pots that can accommodate 6 plantlets, containing 2L of a 0.5 mM CaSO_4_ solution and grown for 24 h under a 16/8 h light/dark regime at 22–26°C, 40–50% relative humidity, 125 μE m^–2^ s^–1^ light intensity. For the experiments under drought and hypoxic conditions, seedlings were successively grown in a diluted nutrient solution containing 100 μM MgSO_4_, 5 μM KCl, 200 μM K_2_SO_4_, 175 μM KH_2_PO_4_, 400 μM CaSO_4_, 25 μM NH_4_H_2_PO_4_, 2.5 μM H_3_BO_3_, 0.2 μM MnSO_4_, 0.2 μM ZnSO_4_, 0.05 μM CuSO_4_, 0.05 μM NaMoO_4_, 2 μM Fe-EDTA and supplemented with either protein hydrolysates (SICIT Group) or inorganic nitrogen (NH_4_H_2_PO_4_) (Santi et al., 2017). The protein hydrolysate, a liquid formulation obtained from the hydrolysis of cow connective tissue, contains 32.3% (w/w) total carbon (C), 12.3% (w/w) total nitrogen (N), 10.9% (w/w) organic N of which 68.5% (w/w) total amino acids and 12.4% (w/w) free amino acids. The amino acids composition and the mineral ion concentrations are summarized in Supplementary Tables 1, 2. Drought stress was obtained following the method described by Han et al. (2017). The plants were grown in an aerated nutrient solution supplemented with 15% w/w of polyethylene glycol (PEG, MW6000), root growth as well as leaf fresh and dry weight (Supplementary Figure 1) was recorded after 9 days of treatment. To study the effects of PH under hypoxic stress, maize seedlings were grown in the above described nutrient solution interrupting air insufflation (see also Results section for detailed description of the experimental conditions). For the experiments on Fe deprivation and supply, seedlings were grown in 2L pots in a aerated Fe-deprived nutrient solution (Sega et al., 2020) containing 2 mM Ca(NO_3_)_2_, 500 μM MgSO_4_, 100 μM KCl, 700 μM K_2_SO_4_, 100 μM KH_2_PO_4_, 10 μM H_3_BO_3_, 0.5 μM MnSO_4_, 0.5 μM ZnSO_4_, 0.5 μM CuSO_4_, 0.01 μM (NH_4_)_6_Mo_7_O_24_ for 7 days. Afterwards, seedlings were transferred in the nutrient solution containing 20 μM Fe, as either FeEDTA (−Fe/+FeEDTA), or FeCl_3_ supplemented with 0.1 mL L^–1^ PH (−Fe/+FeBio) or with the equivalent amount of N supplied (−Fe/+FeCl_3_) as inorganic nitrogen (NH_4_H_2_PO_4_). Control plants were grown in the nutrient solution containing FeEDTA 20 μM for 14 days (+FeEDTA) or in the nutrient solution deprived of Fe with the addition of 14.3 mgL^–1^ of N (−Fe/−Fe). Primary, seminal and lateral root total length and surface area were measured with WinRHIZO^TM^ scanner and automated software (Arsenault et al., 1995).

### Macro- and Micro-Nutrients Quantification

The Fe concentration in the leaf samples and in the PH product was quantified by ICP-MS analysis. For the analysis dried leaf samples and the PH product (about 5 mg) were weighted and digested in a TFM micro-sampling insert using 350 μl of 69% ultrapure HNO_3_. The inserts were put into a 100-ml oven vessel containing 10 ml of water (milliQ, 18.2 M cm) and 1 ml of 30% H_2_O_2_. As reference we digested 5 mg of NIST 1515 (apple leaves). The digestion of the samples was performed using a microwave oven (Milestone StartDR microwave). A 20-min ramping period was used to reach a digestion temperature of 180°C. The temperature was thereupon maintained for 20 min. At the end, sample were diluted with water (milliQ, 18.2 M cm) to a final concentration of 2% HNO_3_. Multi-elemental analysis was carried out using the Agilent 7500cx ICP-MS (Agilent). Each macro- and micronutrient was quantified using a multi-element standard solution. For the assessment of leaf Fe concentration, we collected three biological samples, each derived from a pool of 4 leaves, and two technical replicates for each measurement. For the analysis of PH mineral content, the mean values were calculated using three technical replicates.

### SPAD Measurements

A SPAD-502 Plus Chlorophyll meter^®^ (Konica Minolta) was used to acquire SPAD values of the third fully expanded leaf of each plant. The values were distinctly taken at the top or at the mid part of the leaves. Measurements were acquired immediately before Fe was supplied in the hydroponic solution, and two, four, and 7 days after that moment. Values from nine to twelve leaves per experimental condition were sampled and averaged to a single SPAD value for each treatment.

### Circular Dichroism Spectral Measurement

Circular dichroism (CD) spectra were recorded with a JASCO J-1500 spectropolarimeter (Japan Spectroscopic Co., Tokyo, Japan), using a quartz cell of 1 mm path length at 25°C. CD spectra were scanned in the far-ultraviolet range from 190 to 250 nm. Values were measured at an interval of 1 nm, and the spectra obtained were the average of 5 reads. The biostimulant was diluted in distilled water to get a total amount of nitrogen of 71.5 mg L^–1^. FeCl_3_ was added to the biostimulant in aqueous solutions at different concentrations [(μM) 0, 50, 100, 150, 200, 250]. Spectra of FeCl_3_ dissolved in distilled water without the biostimulant, at different concentrations, were used as blank reference. Final spectra resulted from the subtraction of their respective blanks and subsequent smoothing. The CD data were expressed in terms of molar ellipticity, [θ], in deg cm^2^ dmol^–1^ and were calculated referring to a Mean Residual Weight (MRW) fixed at 106.5 g mol^–1^.

### Quantitative RT-PCR Analysis

Total RNA was extracted from root samples, using the Spectrum^TM^ Plant Total RNA kit (Sigma-Aldrich, St. Louis, MO, United States) and quantified using NanoDrop^TM^ 1000 (Thermo Scientific, Waltham, MA, United States). For RNA extraction we used three biological samples, each consisting of the roots of four plants. DNase I treatment was carried out for each sample using one microgram of total RNA and the RQ1 RNase-Free DNase (Promega, Madison, WI, United States) according to the manufacturer’s procedure. DNase-treated samples were then used to synthetized cDNA with the ImProm-II Reverse Transcription System (Promega, Madison, WI, United States). Real-time RT-PCR experiments were performed with the Fast SYBR^®^ Green Master Mix (Thermo Fisher Scientific) on the QuantStudio 3 Real Time-PCR (Thermo Fisher Scientific) according to the manufacturer’s protocols. The reactions were carried out in a volume of 10 μL with a final primer concentration equal to 350 nM and 1 μL of a 1:3 solution of cDNA as template. The following thermal profile was set-up: 95°C for 20 s, 40 cycle of 95°C for 3 s and 60°C for 30 s. Two housekeeping transcripts were employed, one encoding an ubiquitin-conjugating enzyme (GRMZM2G027378_T01) and the other an unknown protein (GRMZM2G047204_T01), respectively. The analysis of the expression of *ZmIRT1* and *ZmYS1* was carried out with primers used by Nozoye et al. (2013) whilst for the expression of *ZmTOM1* the primers were designed accordingly to Chan-Rodriguez and Walker (2018). The sequences of all primers were reported in Supplementary Table 3.

The PCR reaction efficiencies were calculated with the LinRegPCR program (Ramakers et al., 2003). Mean normalized expression (MNE) (Simon, 2003) was calculated for each transcript and each sample using the two housekeeping transcripts separately. A mean MNE value was determined using a geometric mean of the two MNE values obtained for each transcript and each sample (Vandesompele et al., 2002).

### Statistical Analysis

Statistical analysis was performed using GraphPad Prism version 5.03 software. Student’s *t*-test for unpaired data was applied when two independent variables were compared as is the case of data shown in Figures, and a *p* value < 0.05 was accepted as statistically significant. Data are presented in the figures as mean and standard error of the mean.

## Results

### The Stimulation of Root Growth Induced by PH Is Not Affected by Drought Conditions

**FIGURE 1:**
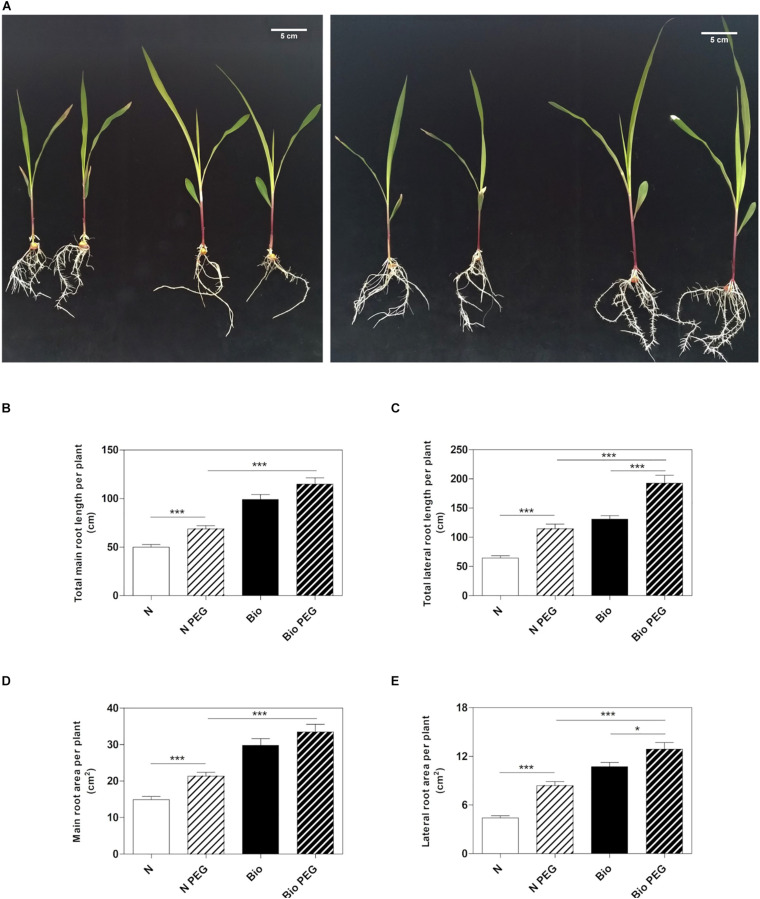
Effects of the PH on root growth of maize plants subjected to drought stress. Maize plantlets grown with or without 15% PEG in the nutrient solution after 9 days of treatment with either 0.1 mL L^–1^ PH (Bio) or with the equivalent amount of total N (14.3 mg L^–1^), supplied as inorganic nitrogen (N) **(A)**. Total seminal and primary root length **(B)**, total lateral root length **(C)**, total seminal and primary root area **(D)** and lateral root area of the maize plantlets treated as described above **(E)**. Root length and root area were measured with WinRHIZO^TM^ software. Mean values per plant are reported. Bars represent the standard error (SEM) (*n* = 21), Student’s *t*-test was applied, *p < 0.05; ***p < 0.001.

To investigate whether the PH could mitigate drought stress, we grew maize seedlings from the emergence of the primary root in a diluted nutrient solution supplemented with the PH (Bio) or inorganic nitrogen (NH_4_H_2_PO_4_; N) and 15% w/w of polyethylene glycol (PEG, MW6000) (Figure 1A). After 9 days of treatment, we analyzed the length and surface area of the roots. Consistently with previous results (Santi et al., 2017), the plants grown in the presence of the protein hydrolysate under normal condition had longer main and lateral roots compared to inorganic N-treated plants (Figures 1B,C). The improved root growth confers to Bio-treated plants also an extended absorption surface (Figures 1D,E). Thus, we confirmed that the PH promotes the root apparatus development when water is abundantly available. Under drought conditions obtained by using 15% PEG, plants treated with N showed a significant increase in both main and lateral root length (+37.6 and +78.1%, respectively) and area (+43.1 and +90.1%, respectively) with respect to non-stressed plants. In plants treated with Bio, drought conditions did not lead to statistically significant increase in the main root growth, while lateral roots length and area (+51.0 and +23.2%) were augmented although to a lesser extent as compared to the N-treated plants. Notably, under drought stress, plants treated with the PH maintained a significantly higher root growth compared to the plants supplied with inorganic nitrogen (main root length: +67.0%; lateral root length: +73.0%; main root area: +61.4%; lateral root area: +57.8%).

### The PH Counteracts the Inhibitory Effects of Hypoxia on Root Growth

**FIGURE 2:**
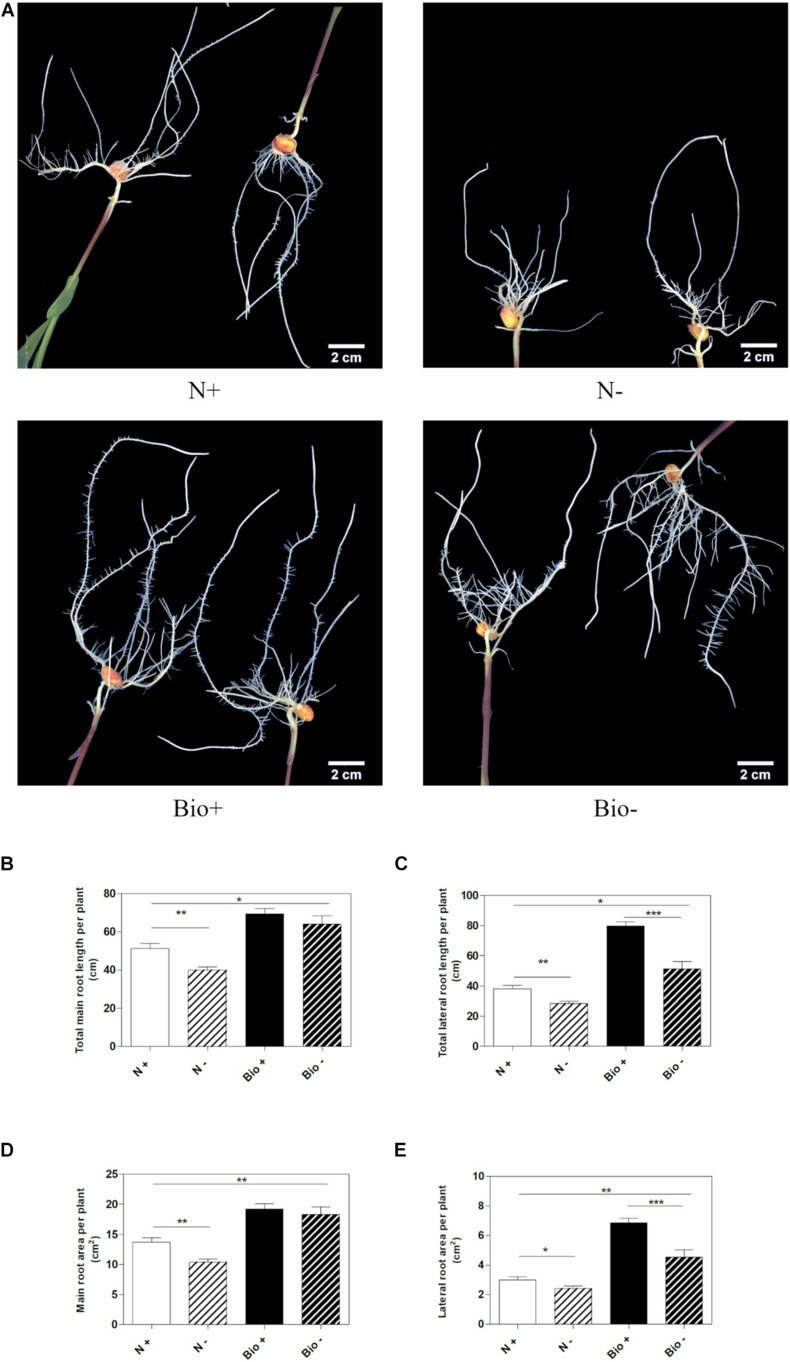
Effects of the PH on root growth of maize plants subjected to hypoxic stress. Maize plantlets grown with (+) or without (–) air insufflation in the nutrient solution after 4 days of treatment with either 0.1 mL L^–1^ PH (Bio) or with the equivalent amount of total N (14.3 mg L^–1^), supplied as inorganic nitrogen (N) **(A)**. Total seminal and primary root length **(B)**, total lateral root length **(C)**, total seminal and primary root area **(D)**, and lateral root area **(E)**, of the maize plantlets treated as described above. Root length and root area were measured with WinRHIZO^TM^ software. Mean values per plant are reported. Bars represent the standard error (SEM) (*n* ≥ 22), Student’s *t*-test was applied, *p < 0.05; **p < 0.01; ***p < 0.001.

To establish whether the PH could alleviate hypoxic stress, maize seedlings were grown hydroponically in a diluted nutrient solution supplemented with the PH (Bio) or inorganic nitrogen (NH_4_H_2_PO_4_; N). Control plants (N+; Bio+) were grown in pots with air insufflation, while hypoxic stress was imposed (N−; Bio−) by interrupting air flow. The plants were grown for 4 days after the emergence of the primary root, then, roots length and area were analyzed (Figure 2A). Under hypoxic conditions, plants treated with inorganic nitrogen (N−) showed a statistically significant reduction of main and lateral root length (−22.1 and −25.6%, respectively) (Figures 2B,C) and area (−24.2 and −19.5%, respectively) compared to the control (N+) (Figures 2D,E). The hypoxic stress in the Bio-treated plants resulted in the inhibition of lateral root growth and surface area (−35.6 and −33.7%), a bit higher than what was observed in N-treated plants. However, the PH treatment (Bio−) alleviated the effects of hypoxia on the main roots, since their length and surface area did not differ from those of Bio-treated control plants (Bio+). Overall, under hypoxic conditions, the root apparatus of Bio-treated plants (Bio−) was more developed than that of N-treated plants even when grown under sufficient oxygen supply (N+).

**FIGURE 3:**
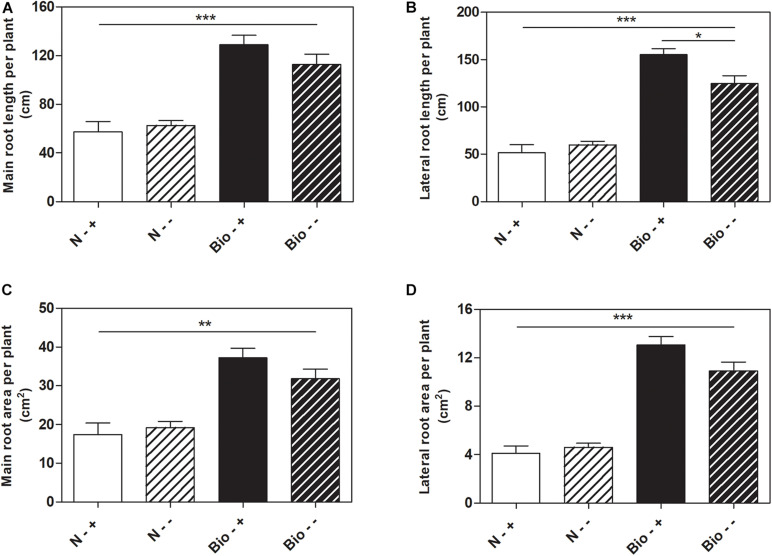
Effects of the PH on root growth of maize plants after recovery from hypoxic stress. Total seminal and primary root length **(A)**, lateral root length **(B)**, total seminal and primary root area **(C)**, lateral root area **(D)**, of maize seedlings treated with 0.1 mL L^–1^ PH (Bio) and seedlings treated with equivalent amounts of total N (14.3 mg L^–1^), supplied as inorganic nitrogen (N). The seedlings were hydroponically grown under hypoxia for 5 days, and then kept under normal air flow or hypoxic stress for further 4 days. Root length and root area were measured with WinRHIZO^TM^ software. Mean values per plant are reported. Bars represent the standard error (SEM) (*n* ≥ 7), Student’s *t*-test was applied, *p < 0.05; **p < 0.01; ***p < 0.001.

Further analyses were conducted to test whether the treatment with PH could favor root recovery from hypoxic stress. We therefore set up a new experiment in which plants cultivated with either the PH or inorganic nitrogen were grown hypoxically for 4 days and then supplied with air or maintained under hypoxia for further 5 days. Plants treated with inorganic nitrogen and grown for 9 days under hypoxic stress (N−−) grew stunted compared to those treated with the PH (Bio−−) (Bio−− vs. N−−: main root length: +80.1%; lateral root length: +108.1%; main root area: +65.6%; lateral root area: +136.8%; *p* < 0.001 for all the comparisons) (Figures 3A–D). When plants treated with inorganic nitrogen were supplied with air (N−+), the root growth did not ameliorate significantly compared to that of the oxygen depleted plants (N−−). Conversely, plants treated with the PH and supplied with air (Bio−+) showed a statistically significant increase (+24.4%) in lateral root length compared to those maintained in hypoxia (Bio−−). As for the other growth parameters, the trend observed was similar (Bio−+ vs. Bio−− seminal root length: +14.5%; seminal root area: +16.9%; lateral root area: +19.6%). Thus, the treatment with the PH showed beneficial effects on root growth under hypoxic stress and most probably facilitate the recovery once the plants return to normal oxygen regimen.

### The Presence of PH in the Growing Solution Favors the Recovery From Fe Deficiency

**FIGURE 4:**
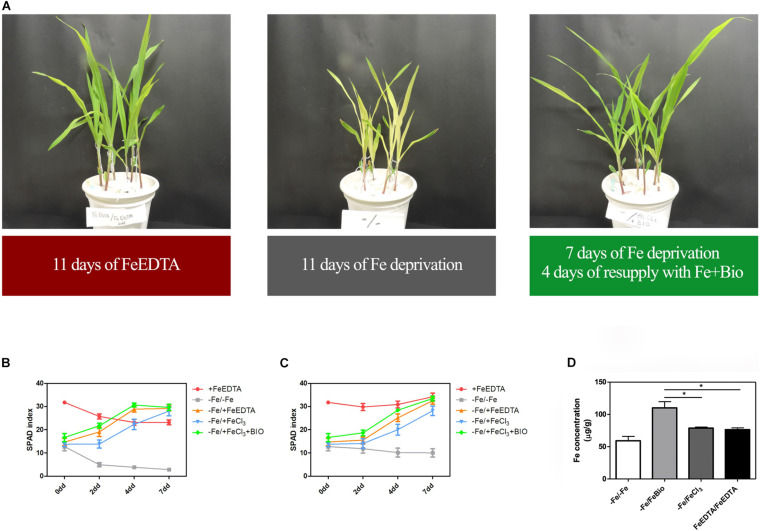
Effects of PH on recovery from Fe deprivation. Maize plantlets grown in nutrient solutions with FeEDTA for 11 days, without any Fe source for 11 days, or without Fe for 7 days and then supplied with FeCl_3_ mixed with the biostimulant for further 4 days **(A)**. SPAD values progression in leaves of maize seedlings treated with the PH (Bio) during recovery from Fe deprivation. Maize plantlets were grown in a nutrient solution containing FeEDTA 20 μM for 14 days (+ FeEDTA), or in an Fe-depleted nutrient solution (-Fe) for 7 days, and supplied with 20 μM Fe as either FeEDTA (–Fe/ + FeEDTA), FeCl_3_ (–Fe/ + FeCl_3_) or FeCl_3_ supplemented with 0.1 mL L^–1^ PH (–Fe/ + FeBio). A group of plants were maintained in the nutrient solution deprived of Fe with the addition of 14.3 mg L^–1^ of N supplied as inorganic nitrogen (–Fe/–Fe). SPAD values were taken at the top **(B)** and mid **(C)** part of the leaf. Mean values are reported. Bars represent the standard error (SEM) (*n* ≥ 9). Fe concentration in maize leaves subjected to different treatments during recovery from Fe deprivation **(D)**. Values of Fe concentration assessed via ICP-MS in maize leaves harvested after 7 days of Fe supply. Mean values are reported. Bars represent the standard error (SEM) (*n* = 3), Student’s *t*-test was applied, *p < 0.05.

To investigate whether the PH can favor plant Fe uptake after starvation, maize seedlings were grown in an Fe-depleted solution for 7 days, and then Fe was supplied to a concentration of 20 μM as Fe chelated with EDTA, FeCl_3_, or FeCl_3_ previously mixed with the PH (Figures 4A–C). Control plants were grown in a nutrient solution containing 20 μM FeEDTA (+FeEDTA), or in the absence of Fe throughout the experiment (−Fe/−Fe). We monitored the pH of the different nutrient solutions every day during Fe supply. In all the treatments the pH was favorable for Fe uptake and showed little variation among the different solutions, ranging from 5.2 to 5.3 at first day of supply to 5.9–6.2 at the end of the experiment.

At the 2nd day after the Fe supply, plants treated with the PH performed as well as the plants treated with FeEDTA (−/+FeEDTA), and significantly better than those grown in the solution containing the Fe salt only (+56.9%) if we consider the SPAD values detected in the middle part of the leaf, while no significant differences were observed in the top part (Figures 4B,C and Table 1). On the 4th day, SPAD values of plants supplied with Fe mixed with the PH were significantly higher than those of plants treated with FeCl_3_ alone (TOP leaf: +42.0%, MID leaf: +37.5%) and similar to those of plants supplied with FeEDTA (Figures 4A–C). At the 7th day, maize grown with FeCl_3_ reached mid-leaf SPAD values comparable to those of FeCl_3_ plus protein PH, while the top-leaf values were still higher for those treated with the PH (+18.7%). Overall, during the supply, the plants treated with the mix of Fe and the PH were the fastest to overcome foliar chlorosis, reaching in only 4 days similar top-leaf SPAD values than those of the plants which did not undergo Fe starvation. Furthermore, these values were even increased in the mid-leaf (+32.3%). Similarly, the plants supplied with FeEDTA were able to recover from the chlorosis at the 4th day on the mid-leaf (+24.6%). However, they reached top-leaf SPAD values similar to the positive control just at the 7th day of supply. The worst performing plants were those supplied with FeCl_3_: although able to reach the mid-leaf SPAD values of the positive control at the 4th day, they did not show an increase conversely to the other two theses; moreover, they could not equal the performance of the control plants at the top of the leaf (−17.5%). The analysis of the plants maintained in FeEDTA for the entire length of the experiment revealed a progressive decrease in the mid leaf SPAD values which was probably due to the rapid growth of the plants under optimal Fe conditions.

**TABLE 1:** Comparison of SPAD values in maize seedling subjected to different treatments during recovery from Fe deprivation.

Vs.		MID			TOP		
		
		FeEDTA/FeEDTA	−Fe/FeEDTA	−Fe/FeCl_3_	FeEDTA/FeEDTA	−Fe/FeEDTA	−Fe/FeCl_3_
2°day	−Fe/FeEDTA	−25.9%(**)			−47.9% (***)		
	−Fe/FeCl_3_	−46.2%(***)	−27.3%n.s.		−52.7%(***)	−9.3%n.s.	
	−Fe/FeBio	−15.5%(*)	14.0%n.s.	56.9%(**)	−37.4%(***)	20.1%n.s.	32.4%n.s.
4°day	−Fe/FeEDTA	24.6%(**)			−18.5%(*)		
	−Fe/FeCl_3_	−3.8%n.s.	−22.8%(*)		−35.1%(***)	−20.4%n.s.	
	−Fe/FeBio	32.3%(***)	6.2%n.s.	37.5%(**)	−7.9%n.s.	13.0%n.s.	42.0%(***)
7°day	−Fe/FeEDTA	26.0%(***)			−4.9%n.s.		
	−Fe/FeCl_3_	21.0%(*)	−4.0%n.s.		−17.5%(*)	−13.3%n.s.	
	−Fe/FeBio	28.0%(**)	1.5%n.s.	5.8%n.s.	−2.1%n.s.	2.9%n.s.	18.7%(*)

Percentage variations of SPAD values recorded at mid-leaf (MID) and top-leaf (TOP) after 2, 4 or 7 days of Fe supply. Student’s *t*-test was applied, *p < 0.05; **p < 0.01; ***p < 0.001.

The positive effect of PH on Fe-deprivation recovery was confirmed by the analyses of Fe concentration in maize leaves (Figure 4D). In fact, in plants supplied with Fe together with the PH for 7 days the concentration of Fe was higher than that of plants grown with FeEDTA or those supplied with FeCl_3_. These latter plants were able to reach a concentration comparable to that of the positive control plants, but significantly lower than those grown with Fe and the PH (−25.34%).

To exclude any direct contribution of the PH on plant Fe uptake, we grew Fe-deprived plants in the nutrient solution with the addition of the PH (0.1 mL L^–1^) alone. We have compared the SPAD values measured for seven consecutively days in the leaves of plants grown without Fe and plants grown without Fe but with the addition of PH. The values recorded were similar in both treatments (−Fe and −Fe + PH) ranging from 3.5 to 10, which are typical of chlorotic maize leaves. In addition, we assessed the concentration of Fe in the product (Supplementary Table 2). At our experimental condition (1:10,000 PH dilution) the Fe supplied by PH was calculated to be at a concentration of 0.032 μM, therefore irrelevant compared to that presents in the nutrient solution.

### The PH Interacts With Fe Ions

**FIGURE 5:**
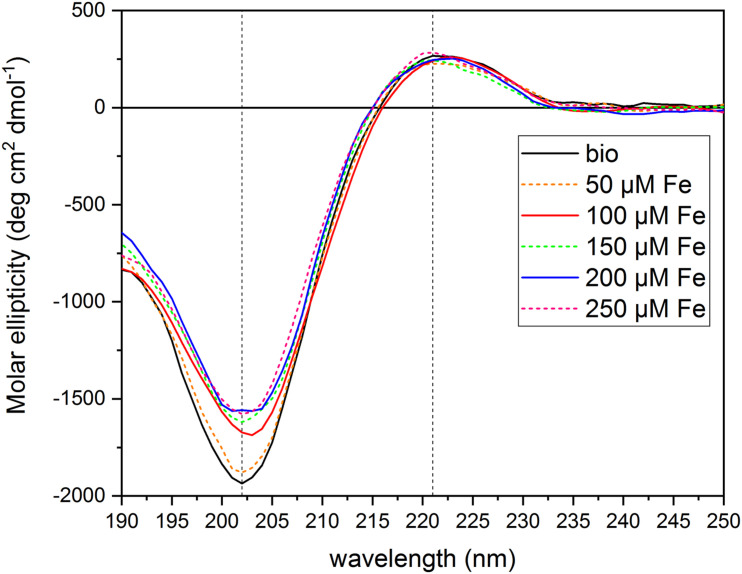
Circular dichroism spectra of protein hydrolysate dissolved in aqueous solutions with FeCl_3_ at different concentrations. The figure shows the CD spectra of the PH diluted in aqueous solutions ([N] = 71.5 mg L^–1^) with different FeCl_3_ concentrations [(μM) 0, 50, 100, 150, 200, 250]. Dashed lines mark 202 and 221 nm wavelengths. Molar ellipticity was calculated referring to a Mean Residual Weight (MRW) fixed at 106.5 g moL^–1^.

To test the hypothesis that the PH under study can act as an Fe chelator, we evaluated the interaction of the PH with Fe ions by circular dichroism. We collected the CD spectrum of the PH itself dissolved in water ([N] = 71.5 mg L^–1^), and then the spectra of the PH mixed with FeCl_3_ at six different concentrations [(μM) 0, 50, 100, 150, 200, 250)]. We set the PH concentration to a total amount of N of 71.75 mg L^–1^ and the FeCl_3_ central concentration of the range to 100 μM, in order to obtain the same ratio between the Fe and the PH concentrations used in the hydroponic system for the Fe supply experiments. The PH CD spectrum obtained (Figure 5) is an intermediate between the typical soluble type II collagen spectrum, which features two characteristic peaks, a large negative one at 197 nm, and a small positive one at 220 nm, and the polyproline spectrum, which displays the large negative peak shifted at 206 nm, and the small positive one at 228 nm (Lopes et al., 2014). For instance, the CD spectrum of the PH presents two peaks one at 202 nm and the other at 221 nm, implying that for this product the chemical hydrolysis does not alter considerably the capability of the matrix to form polyproline II (PPII)-type structures. The CD spectra of the PH mixed with Fe showed a dose-dependent reduction of the peak at 202 nm compared to the reference spectrum of the PH alone (Supplementary Table 4), while the peak at 221 nm did not show major alterations when Fe is added (data not shown). At the lowest Fe concentration, 50 μM, a slight increase of the molar ellipticity value at 202 nm was observed; adding 100 μM Fe we found a greater increase; for concentrations higher than 150 μM, the molar ellipticity value remained almost unaltered, suggesting that the interaction between the PH and the Fe ions was saturated at 150 μM. These findings suggest that the PH PPII-type structure undergoes a slight conformational change in the secondary structure when Fe ions are present in the solutions. The helix is able to interact with the Fe ions, that are therefore responsible for the increase in the molar ellipticity values, although they do not destabilize the structure to the extent of denaturation (Lopes et al., 2014).

### The PH Induces Transcriptional Changes in Maize Roots During Recovery From Fe Deficiency

**FIGURE 6:**
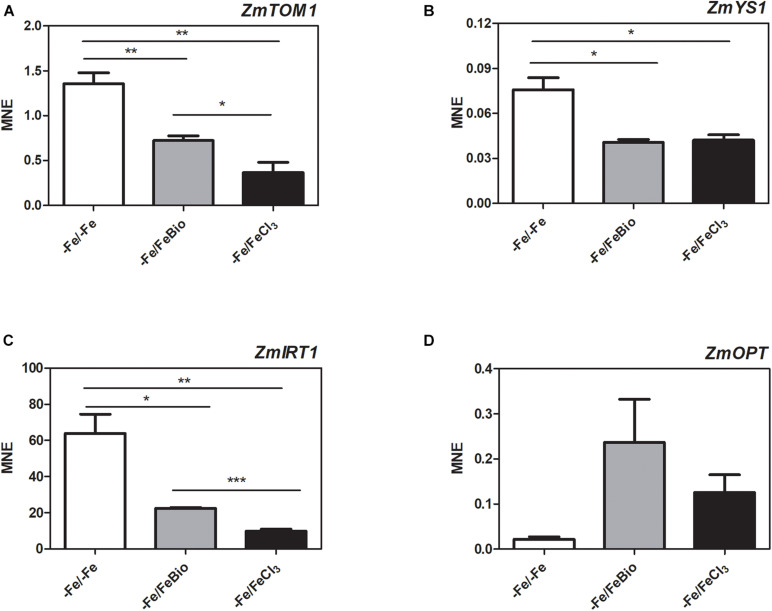
Quantitative RT-PCR analysis of genes involved in Fe uptake in maize roots. Relative transcript level, calculated as Mean normalized expression (MNE) of *ZmTOM1* **(A)**, *ZmYS1* **(B)**, *ZmIRT1* **(C)** and *ZmOPT* **(D)** was assayed in roots of plants grown under Fe-deprivation conditions (–Fe/–Fe) and after 24 h Fe-resupply either as FeCl_3_ (–Fe/ + FeCl_3_) or FeCl_3_ plus PH (–Fe/FeBio). The values are the mean of three biological replicates. Bars represent the standard error (SEM). Student’s *t*-test was applied, *p < 0.05; **p < 0.01; ***p < 0.001.

We carried out a transcriptional analysis on the effects of the PH in maize roots during Fe supply in order to shed light on the mechanisms of action that favors recovery from Fe deficiency. We studied the expression of key genes involved in Fe uptake (*ZmTOM1*, *ZmYS1*, and *ZmIRT1*) and in peptide transport (*ZmOPT ids.* GRMZM2G152555_T01, Zm00001d031287_T001) in roots subjected for 7 days to Fe-deprivation (−Fe) and supplied for 24 h with either 20 μM FeCl_3_ (−Fe/+FeCl_3_) or FeCl_3_ plus PH (−Fe/+FeBio) in comparison to roots of plants always grown under Fe deficiency (7 days plus 24 h; −Fe/−Fe) (Figure 6). *ZmTOM1* codes for an efflux transporter of mugineic acid family phytosiderophores and ZmYS1 codes for a proton-coupled symporter of metals chelated to phytosiderophore and to nicotianamine (Schaaf et al., 2004) and both play a key role in Strategy II of Fe acquisition employed by the graminaceous species such as maize (Kobayashi and Nishizawa, 2012). On the other hand, ZmIRT1 protein is a Fe(II) transporter involved in the Strategy I (reduction strategy). These three proteins (ZmTOM1 ZmYS1, ZmIRT1) have been shown to increase their expression level in maize roots under Fe deficiency (Li et al., 2013; Nozoye et al., 2013; Wairich et al., 2019). We clearly observed this effect after 24 h of FeCl_3_ supply (Figures 6A–C). In fact, Fe supply caused a strong reduction of the transcript levels of *ZmTOM1*, *ZmYS1*, and *ZmIRT1* with respect to the roots of maize plants continuously grown under Fe deficiency. In the roots supplied with FeCl_3_ in the presence of the PH, the expression level of *ZmTOM1* and *ZmIRT1* was significantly higher than the expression measured with FeCl_3_ alone, but still lower than the transcript levels of Fe-deprived roots (Figures 6A,C). We did not observe any significant difference in the expression level of *ZmYS1* between roots supplied with FeCl_3_ in presence and absence of PH (Figure 6B).

In order to investigate the involvement of peptide transporters in the mechanisms that favors Fe recovery in plants treated with the PH, we analyzed the expression of the *ZmOPT*, a gene found to be responsive to the PH treatment (Santi et al., 2017). ZmOPT might be involved in mediating the uptake of Fe bound to peptides. The expression of *ZmOPT* showed an increasing tendency in both roots treated with FeCl_3_ and FeCl_3_ plus the PH, this trend appears more pronounced in the presence of the PH (Figure 6D).

Overall, concerning the modulation of genes involved in response to Fe deficiency, these results indicate that the roots supplied with FeCl_3_ plus the PH displayed an intermediate behavior between Fe-deprived roots and roots supplied with FeCl_3_ alone.

## Discussion

Biostimulants represent a vast category of products used in the agricultural practice to increase crop productivity, nutrient acquisition and stress resistance. They include natural products of animal and plant origin as well as microorganisms. Due to the generally complex nature of the biostimulants, often composed by a mixture of different molecules, it is very difficult to identify the active components responsible for the beneficial effects on crops (Yakhin et al., 2017). On the other hand, the presence of different active biomolecules in a single product can widen the physiological processes that can be targeted. This can explain the various beneficial effects that can be obtained by a single biostimulants.

The scientific research on biostimulants should be focused on two main targets: (i) to identify the bioactive components of a product (ii) to test the activity of the product on different physiological processes and under different environmental conditions. Concerning the first point, the possibility to find out the main active molecules can allow, on one hand to use a reductionist approach for studying the mechanism of action of the biostimulant, and, on the other hand, to optimize the products at the industrial level. This approach appears feasible only with certain categories of biostimulants (e.g., protein hydrolysate obtained from a tissue with few abundant proteins), whereas quite difficult for biostimulants originated from complex matrix (e.g., seaweed extracts). The second goal is important for defining the product claims. The claims are usually based on the results from experiments conducted under green house or field conditions monitoring a single plant trait, principally crop growth or product quality. It is possible therefore that in many cases other beneficial effects can be disregarded. Experiments carried out under laboratory conditions can be used to easily test the effects of biostimulants on a great variety of physiological parameters and under different environmental conditions to obtain information on the potential uses of a product. The set-up of the experiments must be carefully planned to assure reproducibility, for instance the assessment of the biological variability and the choice of proper controls appear crucial. In this work, we have planned a series of laboratory experiments to test whether a collagen-based PH has the capacity to exert beneficial effects on plants subjected to different types of stress. The crop used was maize because in a previous work we demonstrated the positive effects of the collagen-based PH on maize root growth and mineral ion uptake after few days of application (Santi et al., 2017). The plants were grown hydroponically in an aerated nutrient solution supplied with the PH. The use of hydroponic culture has several advantages; for instance, it permits the precise control of the medium composition, the possibility to study the response to deprivation or excess of a single mineral nutrient and also the simulation of various stress conditions. A very critical issue in evaluating the effects of a biostimulant is also the choice of a proper control, in our case as the biostimulant object of the study was a PH, we added to the control nutrient solution NH_4_^+^ to reach the same amount of total N supplied with the PH. In this way we also proved that the effects of the biostimulant are not due to N fertilization. We chose to assess the potential activity of the collagen-based PH as multi-stress protectant against three different types of abiotic stresses: drought, hypoxia and Fe starvation and we analyzed the effects on growth during the first days after germination, a phase that can be critical for the plant emergence after sowing.

Our results confirmed the observations made by Trevisan et al. (2019) about the capacity of the PH to protect the root apparatus under hypoxic conditions and extended the analysis to the advantages that the presence of PH supplied in the medium can bring during the recovery from the stress (Trevisan et al., 2019). Indeed, when after 4 days of hypoxia the plants returned to the normal air insufflation, the presence of the PH in the medium rapidly stimulated the resumption of root growth whereas untreated roots did not seem to recover.

To our knowledge, the effects of collagen-derived PH on plants subjected to water stresses has not previously been investigated. To apply water stress conditions, we follow a method described by Han et al. (2017) to generate osmotic stress in maize plants under hydroponic cultivation by adding 15% PEG to the nutrient solution (Han et al., 2017). The response to osmotic stress was similar in N− and PH-treated plants. In both cases we observed a slight decrease in shoot fresh biomass (Supplementary Figure 1 and Figure 1A) and an increase in root growth – mainly in the lateral roots for what concerns the PH-treated plants- leading to a change in shoot/root ratio which is a typical response of plants to water deficit. Although the plant species and the PEG concentration used are different, our results are in accordance with those of Ji et al. (2014), who showed that one of the effects of PEG-mediated osmotic stress in wheat is the induction of lateral root development (Ji et al., 2014). Overall, we showed that the water stress did not alter the positive effects of PH on root growth most probably allowing under longer term a better acquisition of water and nutrients in the PH- treated plants compared to the control ones.

The positive effects of PH on abiotic stress resistance has often been ascribed to its capacity to counteract the oxidative stress (Lucini et al., 2015) mainly through the induction of genes involved in antioxidant defense (Caruso et al., 2019). In this regard, we previously observed that the collagen-based PH compared to other biostimulants such as free amino acids, produces only a moderate alteration of genes related to oxidative stress under normal conditions (Santi et al., 2017) suggesting that it is not itself an oxidative stress elicitor. Interestingly, the collagen-based PH acts on phytohormone homeostasis and signaling, modifying the expression of genes involved in metabolism, transport and signal transduction of gibberellin, cytokinins and auxin (Santi et al., 2017). It is presumable that the effects of the collagen-based PH on the hormone functions could be responsible for the positive effects on root growth also under stress conditions.

Concerning the experiments of recovery after Fe-starvation, we obtained several lines of evidence about the positive effects of the collagen-based PH. The SPAD measurements and Fe concentrations in the leaves clearly demonstrated that the administration of PH mixed to FeCl_3_ confers a faster recovery from deprivation as compared with treatments with FeCl_3_ or FeEDTA. We can hypothesize that the positive role of the PH on Fe accumulation in starved plants is linked to both biological and chemical proprieties of the PH itself. Some interesting insights concerning the structural features of the PH under study and on its interaction with Fe were discovered thanks to the CD spectra analyses. CD has been widely used as a reliable source to study the interactions among biomolecules (Greenfield, 2007), especially proteins, and some works had already highlighted the diagnostic features of collagen, collagen peptides and poly-proline type II (PPII) structures spectra (Piez, 1968; Piez and Sherman, 1970; Adzhubei et al., 2013; Lopes et al., 2014). The PH spectra obtained was extremely reproducible and had the typical shape of the collagen peptides and PPII (type II polyproline helices) reference spectra (Lopes et al., 2014). The similarities between these CD spectra were striking and we can therefore assume that the acid hydrolysis process produces a mixture of short peptides that maintains the PPII secondary structure. Furthermore, CD spectra showed that the collagen-based PH can interact with Fe in a dose-dependent manner suggesting the formation of Fe-PH complexes. Results previously reported by Santi et al. (2017) hinted that the collagen-based PH could improve Fe uptake through a transcriptional reprogramming of genes playing a role in the metal transport processes. With the exception of *ZmYS1*, here we show that the molecular components involved in maize root Fe acquisition (e.g., solubilization and uptake, *ZmTOM1* and *ZmIRT1*, respectively) (Li et al., 2013, 2018; Nozoye et al., 2013; Wairich et al., 2019) are more expressed in PH-treated roots during the FeCl_3_ supply (Figures 6A–C), possibly indicating that the mechanisms of Fe sensing by maize roots are, in this condition, less susceptible to the restored cellular Fe accumulation. Interestingly, a similar behavior was described in Fe-deficient tomato plants supplied with Fe chelated to fulvic acid like water-extractable humic substances, another category of plant biostimulant (Zamboni et al., 2016). Furthermore, we cannot exclude a role of *ZmOPT* in the uptake of Fe-PH complexes, since it showed the highest expression in PH-treated roots although not statistically significant (Figure 6D).

**FIGURE 7:**
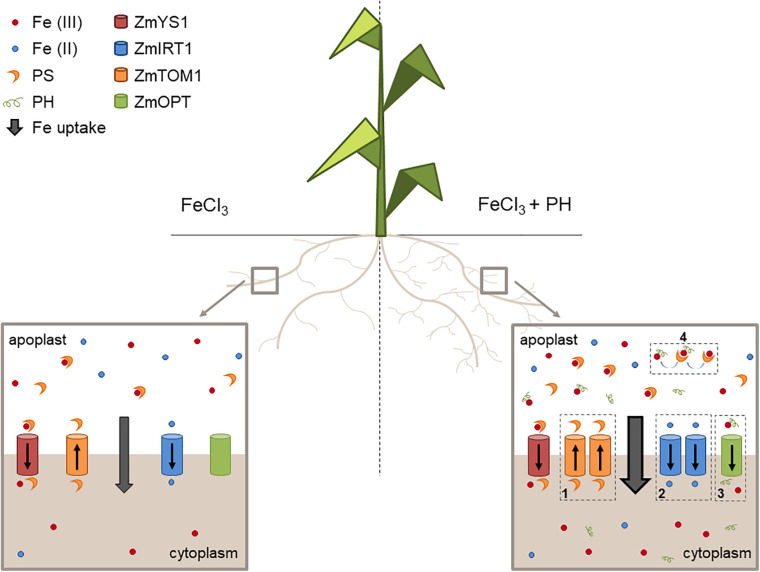
Mechanisms of action of the PH as Fe-deficiency stress mitigator. Schematic representation of the maize root apparatus after the administration of Fe in the absence (left) or presence (right) of the PH. We show that PH increases the lateral root length (Santi et al., 2017) thus favoring soil exploration for nutrients. In addition, PH treatment maintains a higher expression of genes encoding (1) the transporter involved in PS efflux (ZmTOM1) and (2) the transporter of Fe(II) (ZmIRT1), possibly reduced by plasma membrane Fe reductase activities (not shown in the scheme). The PH tends to increase the expression of (3) putative Fe-peptide transporter (ZmOPT). The PH can also chelate Fe and facilitate the exchange of Fe to the PSs (4).

We propose that the PH role as Fe deficiency stress mitigator might be explained with a combination of possibly synergic chemical and molecular mechanisms leading to an increased uptake and distribution of Fe when Fe is supplied after shortage. In particular, these mechanisms seem to be based on: (1) an enhanced stimulation of phytosiderophore (PS) efflux leading to a higher solubilization of Fe(III); (2) an increased uptake of the reduced form of Fe since the presence of *ZmFRO2* in maize genome has been ascertained (Li et al., 2018); (3) an easier uptake of Fe as a chelated complex with the PH; (4) an increased Fe solubilization due to the presence of PH and an effective ligand exchange process involving Fe-PH and PSs making more efficient the action of these chelators (Figure 7).

In conclusion, we demonstrated that the collagen-based PH under study, not only is a plant growth promoting agent, but it might also be used effectively as hypoxic, drought, and nutrient stress mitigator.

The protective effects cannot be related to a single mechanism of action, but most probably are the result of different activities of the peptides both in the external medium and inside the cells. A further characterization of this matrix could help to shed light on the role of its peptides at the cellular level, considering that many works have showed how small peptides are crucial for plant signaling (Gancheva et al., 2019). For instance, IRON MAN (IMA), CLV3/ESR-RELATE D (CLE) and HYDROXYPROLINE-RICH GLYCOPEPTIDE SYSTEMINS (HypSys) are three important families of peptides, on the range of 5–20 amino acids, involved in very different processes. The members of the former family control iron transport in plants (Grillet et al., 2018), those of the second one are involved in the differentiation of shoot and root meristems (Leasure and He, 2012), while those of the third one are plant protective signal peptides, participating in plant defense reactions (Pearce, 2011).

## Data Availability Statement

The original contributions presented in the study are included in the article/Supplementary Material, further inquiries can be directed to the corresponding author.

## Author Contributions

ZV, TP, and AZ contributed to the conception and design of the study. SA, CS, and DS performed the experiments. SA, DS, AZ, ZV, and TP carried out the analysis of the data. SA and TP draft the manuscript. All authors approved the manuscript.

## Conflict of Interest

The authors declare that the research was conducted in the absence of any commercial or financial relationships that could be construed as a potential conflict of interest.
