# Supplementary material for: Evaluation of the Potential Use of a Collagen-Based Protein Hydrolysate as a Plant Multi-Stress Protectant
Source: Front Plant Sci. 2021 Feb 9;12:600623. doi: 10.3389/fpls.2021.600623 (PMC7899969; doi:10.3389/fpls.2021.600623)
Supplement: Supplementary file 1 [file Data_Sheet_1.docx]

Supplementary Material

# Supplementary Tables

Table S1. **Concentration of mineral elements measured in the PH.** Mean concentration and respective standard deviation of Ca, Co, Cu, Fe, K, Mg, Mn, P and Zn assessed via ICP-MS. Values refer to the concentrations in the non-diluted PH. LLOQ: limits of quantification.

| Mineral element | Concentration (µg/g) | Standard Deviation |
| --- | --- | --- |
| Ca | 518,4591 | 5,705144 |
| Co | 0,066369 | 0,001056 |
| Cu | under LLOQ | |
| Fe | 17,87588 | 0,019852 |
| K | 199,5098 | 0,473483 |
| Mg | under LLOQ | |
| Mn | 0,143994 | 0,00242 |
| P | under LLOQ | |
| Zn | 2,351049 | 0,020807 |

**Table S2. Molar ellipticity values at 202 nm of protein hydrolysate dissolved in aqueous solutions with FeCl_3_ at different concentrations.** The table shows the mean molar ellipticity values at 202 nm of the PH diluted in aqueous solutions (N = 71.5 mg L^-1^) with different FeCl_3_ concentrations (µM 0, 50, 100, 150, 200, 250 µM). Molar ellipticity was calculated referring to a Mean Residual Weight (MRW) fixed at 106.5 g mol^-1^. Errors are presented as standard deviations (n=5).

| **Sample** | Molar ellipticity [*ɵ*] deg cm^2^ dmol^− 1^ | Standard deviation |
| --- | --- | --- |
| **PH** | -1935,47 | ± 58,33 |
| **PH + 50 µM Fe** | -1878,83 | ± 66,57 |
| **PH + 100 µM Fe** | -1673,44 | ± 71,22 |
| **PH + 150 µM Fe** | -1619,94 | ± 64,28 |
| **PH + 200 µM Fe** | -1558,90 | ± 68,56 |
| **PH + 250 µM Fe** | -1578,72 | ± 68,75 |
